# Balance and knee extensibility evaluation of hemiplegic gait using an inertial body sensor network

**DOI:** 10.1186/1475-925X-12-83

**Published:** 2013-08-29

**Authors:** Yanwei Guo, Guoru Zhao, Qianqian Liu, Zhanyong Mei, Kamen Ivanov, Lei Wang

**Affiliations:** 1 Shenzhen Institutes of Advanced Technology, The Shenzhen Key Laboratory for Low-cost Healthcare, Shenzhen University Town, 1068 Xueyuan Avenue, Shenzhen 518055, PR China; 2 Graduate University of Chinese Academy of Sciences, Beijing 10049, PR China; 3 School of Physical Education & Sports Science, South China Normal University, Guangzhou, China

**Keywords:** Gait analysis, Body Sensor Network, Hemiplegic gait, Biomechanics

## Abstract

**Background:**

Most hemiplegic patients have difficulties in their balance and posture control while walking because of the asymmetrical posture and the abnormal body balance. The assessment of rehabilitation of hemiplegic gait is usually made by doctors using clinical scale, but it is difficult and could not be used frequently. It is therefore needed to quantitatively analyze the characteristics of hemiplegic gait. Thus the assessment would be simple, and real-time evaluation of rehabilitation could be carried out.

**Methods:**

Twenty subjects (ten hemiplegic patients, ten normal subjects) were recruited. The subjects walked straight for five meters at their self-selected comfortable speed towards a target line on the floor.

Xsens MTx motion trackers were used for acquiring gestures of body segments to estimate knee joint angles and identify gait cycles. A practical method for data acquisition that does not need to obtain accurate distances between a knee joint and its corresponding sensors is presented.

**Results:**

The results showed that there were significant differences between the two groups in the three nominated angle amplitudes. The mean values of balance level of each parameter in hemiplegic gait and normal gait were: 0.21 versus 0.01, 0.18 versus 0.03, and 0.92 versus 0.03, respectively. The mean values of added angles of each parameter in hemiplegic gait and normal gait were: 74.64 versus 91.31, -76.48 versus −132.4, and 6.77 versus 35.74.

**Conclusions:**

It was concluded that the wearable bio-motion acquisition platform provided a practical approach that was effective in discriminating gait symptoms between hemiplegic and asymptomatic subjects. The extensibility of hemiplegic patients’ lower limbs was significantly lower than that of normal subjects, and the hemiplegic gait had worse balance level compared with normal gait. The effect of rehabilitation training of hemiplegic gait could be quantitatively analyzed.

## Background

Balance impairment is a very common cause of disability in hemiplegic patients [1]. Most hemiplegic patients have difficulties in posture control while walking because of the asymmetrical posture and the abnormal body balance, with reduced knee joint angle and abnormal gait. Previous reports have shown that these patients have an increased risk of falls [2]. Rehabilitation is usually conducted to preserve and recover motor functions, and main focus falls on the assessment and treatment of the gait. The assessment of rehabilitation is usually made by doctors using clinical scale such as Brunnstrom, but this approach is difficult and could not be used frequently. It is therefore needed to quantitatively analyze the characteristics of hemiplegic gait, and find the relation between the characteristics and functional rehabilitation. Thus the assessment would be simple, and real-time evaluation of rehabilitation could be carried out.

Gait analysis is widely used in detecting human walking disorders. There are mainly two gait analysis approaches that have been developed for analyzing human walking. One approach uses marker systems including video based systems, active magnetic trackers, optical marker systems, to get information about human gait movement; however, these systems depend on markers and could not be used outside the laboratory environment. Video based systems usually cause invasion of human privacy and are expensive. The other approach uses body-worn, low-power wearable sensors, such as inertial/magnetic sensor systems, and portable recording systems for long-term ambulatory monitoring. These systems do not depend on markers and allow real-time capturing and analysis of gait parameters over long distance outside the laboratory environment.

Accelerometers and/or gyroscopes have been used to obtain gait parameters [3-11], which can be derived by the integration of angular acceleration or angular velocity. However, data can be distorted by offsets or drifts [12,13]. To eliminate any drift during integration, Morris [14] identified the beginning and the end of gait cycles, and made the signal at the beginning and the end of the cycle coincide. Some researchers [13,15] used accelerometers and gyroscopes fixed on metal plates to measure human joint flexion-extension angles, but they found that the use of metal plates is cumbersome. L.Atallah and Benny Lo et al. [16,17] developed an ear-worn sensor for gait monitoring. Dejnabadi et al. [18] developed a method of measuring joint angle using a combination of accelerometers and gyroscopes by placing a pair of virtual sensors on the adjacent segments at the center of rotation. S. Kobashi et al. [19] used inertial sensors combined with magnetic sensor to estimate 3D knee joint angle. Limitations of the last two methods were that they all need to obtain accurate positions of the physical and virtual sensors to minimize the error.

The method described in this paper uses Xsens MTx motion trackers for acquiring gestures of body segments to estimate knee joint angles and identify gait cycles. It does not need to obtain accurate distances between a knee joint point and its corresponding sensors. Thus our method allows to practically and quantitatively analyze the parameters of hemiplegic gait, and to find the differences of gait parameters between hemiplegic patients and normal subjects.

## Methods

### Platform

Commercial equipment could be used for acquisition of human movement, which has a competitive advantage when applied to non-video monitoring environments. The method described in this paper uses MTx motion trackers (Xsens Technologies B.V., the Netherlands) to acquire the movements of human lower limbs. The MTx is a small and accurate 3DOF inertial orientation tracker which provides drift-free 3D orientation and kinematic data of human body segments: 3D acceleration, 3D rate gyro and 3D Earth-magnetic field. The Xbus Kit (Xsens Technologies B.V., the Netherlands) contains an Xbus Master with Bluetooth wireless link, a wireless receiver and a number of MTx sensor modules. The Xbus Master is a lightweight, portable device that controls multiple MTx modules on the Xbus. Xbus Master and MTx sensor modules are powered by batteries which allow continuous operation for at least 3 h.

### Sensor location

**Figure 1:**
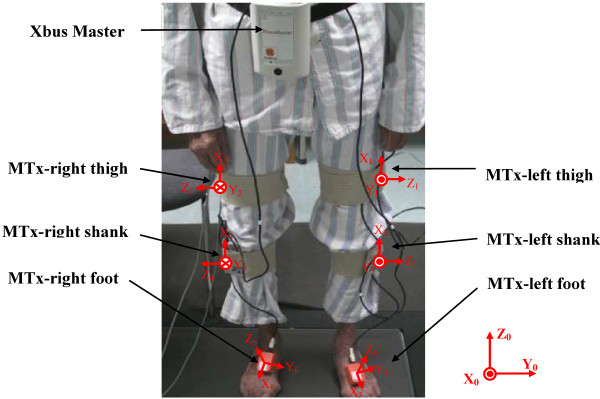
A hemiplegic patient wore the inertial body sensor network (an Xbus Master and six MTx sensors).

Six sensor nodes were used for the experiment (as shown in Figure 1). Four sensors were attached to thigh and shank, on the lateral skin surface near the knee joint and ankle joint, respectively. The other two sensors were attached on the dorsum of feet.

The sensor axes were adjusted in the anterior-posterior plane to accurately measure the motion in the sagittal plane.

The sensors were attached using medical bands. The sensor attachment locations are optimized to reduce the skin movement artifacts based on the clinician’s knowledge and experience [20]. Acceleration, angular rate and magnetic vectors were obtained at sampling frequency of 100 Hz.

### Coordinate system

The initial coordinate system X_0_Y_0_Z_0_ was difined as: we chose the Z-axis to be directed upwards, perpendicular to the horizontal plane and X-axis to be directed forwards, in parallel with the subject’s sagittal plane. The Y-axis was a cross product of X-axis and Z-axis.

The femoral coordinate systems X_1_Y_1_Z_1_ and X_2_Y_2_Z_2_ were defined using three anatomical feature points. The Z-axis was directed towards the lateral epicondyle, connecting it with the medial epicondyle. The X-axis, being perpendicular to the Z-axis, was directed towards the great trochanter. The Y-axis was a cross product of X-axis and Z-axis.

The tibial coordinate systems X_3_Y_3_Z_3_ and X_4_Y_4_Z_4_ were defined by four anatomical feature points. Z-axes were coincided with the Z-axis of the femoral coordinate system. The point To was the middle point between lateral epicondyle and medial epicondyle. The X-axis was defined as the line directed towards the point To, that connects it with the middle point between the lateral malleolus and medial malleolus. Then, the Y-axis was a cross product of X-axis and Y-axis.

The foot coordinate systems X_5_Y_5_Z_5_ and X_6_Y_6_Z_6_ were defined as follows: the Z-axis was perpendicular to each foot plate, with direction upwards; the X-axis was in parallel with each foot plate, with direction forwards; the Y-axis was a cross product of Z-axis and X-axis.

The initial coordinate system and body coordinate systems were defined as described above, using the measured anatomical feature points. All MT sensors’ orientation output values were set to zero when the sensors’ axes were exactly aligned with the axes of the initial system. The rotation transform parameters that are needed to align the coordinate systems of a sensor and a human body segment were employed. The data obtained from the sensors were corrected by the rotation transform parameters to acquire the acceleration and magnetic vectors of the bones.

### Experimental design

Twenty subjects (ten hemiplegic patients, ten normal subjects) were recruited. There were five hemiplegic patients with abnormal left lower limb, and five patients with abnormal right lower limb. The experimental procedures were in accordance with the Declaration of Helsinki and were approved by the ethic committee of Shenzhen Institutes of Advanced Technology. Each subject signed informed consents prior to testing. The group included fifteen males and five females with average age of 58.3±12.85 years. The subjects were asked to stand still for 5 seconds for sensor calibration, then to walk straight for five meters at a self-selected comfortable speed towards a target line on the floor. Each subject performed this procedure three times. The actions of each subject were recorded in real time by a camera.

### Acquisition of gesture data

The classical method to describe a gesture of an object is to use Euler angles, i.e. roll angle, yaw angle and pitch angle. Gestures of an object could be determined by only integration of angular rate data. However, this solution would be prone to drift over time due to the buildup of bias and drift errors. In order to avoid drift, additional complementary sensors must be used. These sensors usually include accelerometers and magnetometers. Measuring the gravity vector using accelerometers allows estimation of orientation relative to the horizontal plane which can be described by roll angle and pitch angle. However, when the object is rotated around the vertical axis, the gravity vector of each axis of the accelerometer will not change. Since accelerometer data could not be used to describe the rotation around the vertical axis, magnetometer is used to measure the local magnetic field vector to determine the orientation relative to the vertical by calculating the angle between object and geomagnetic North Pole. The data from the incorporated sensors is normally fused using Kalman or other complementary filtering algorithm [21].

The rotation angles, which resulted from the object’s rotation from one gesture to another, could be expressed by Euler angles, i.e. the rotation angle of X-axis corresponds to the yaw angle in Euler angles, which could be derived from quaternion (the other classic method to describe a gesture). In this study, the quaternion was directly acquired from the output of Xsens MTx motion trackers.

### Flexion/extension angle

The Z-axis of sensors on the thigh and shank (at the same limb side) were adjusted to be in the same directions. In this way, the rotation angle of thigh and shank in sagittal plane could be regarded as the angle between two sensors in XY plane. Thus, a virtual point was used as a knee joint center, with two virtual lines in parallel with the X-axis of each sensor, respectively. It could be regarded that the sensors on the thigh and shank are moved along the virtual lines towards the knee joint until the both sensor centers coincide. Then the knee joint flexion/extension angle could be described by the angle between two virtual lines.

We defined that the coordinate system of the shank sensor will be used as a reference. Then rotations of the thigh could be described in relation to the shank. Let *q*_*thigh*_ and *q*_*shank*_ represent the quaternion that describes the gesture of sensors on the thigh and shank relative to the initial coordinate system, *q*_*s-t*_ represent the quaternion that describes the gesture of sensor on the thigh relative to the relative coordinate system. The rotation could be described as follows:

$$q_{s-t}=q_{\mathrm{shank}}^{-1}⊗q_{\mathrm{thigh}}$$

The angle amplitude of knee flexion/extension (AK) was defined as the maximum knee flexion/extension throughout one gait cycle.

### Gait cycle

**Figure 2:**
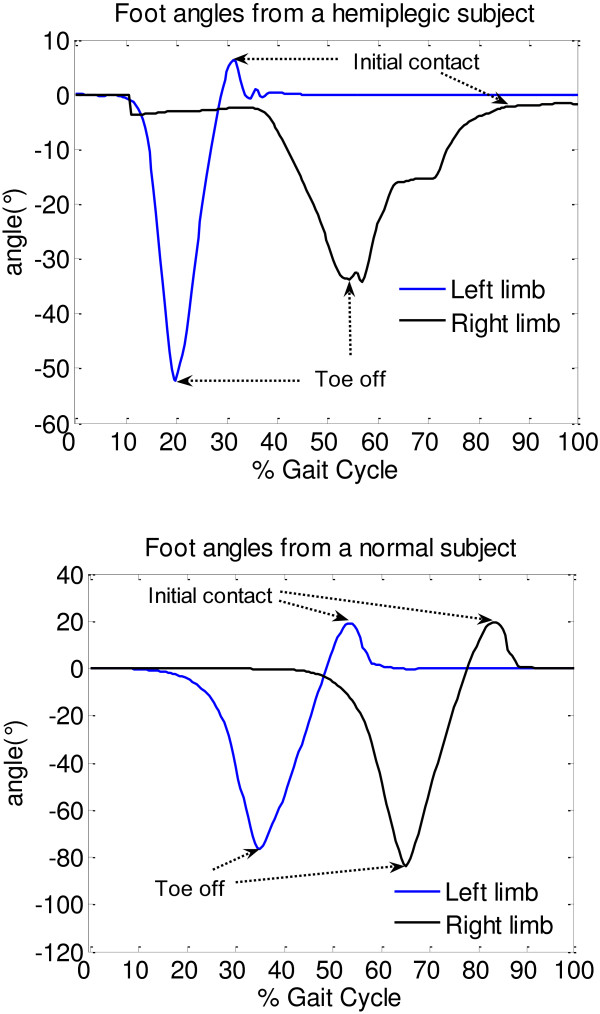
**Gait cycle from a hemiplegic patient (above) and a normal subject (below)**. Z-axis direction of the sensor on foot was taken as positive angle. The initial contact, toe off could be easily identified.

The gait cycle is defined as the time interval between two successive occurrences of one of the repetitive events of walking. There were many researches on gait parameter identification that used inertial sensors. Katia Turcot et al. [22] used maximal and minimal values of acceleration in gait cycle to identify the events ‘initial contact’ and ‘toe off’. Arash et al. [23] used the positive and negative peak angular velocities from sensor on the shank to detect gait cycles and estimate temporal parameters of gait. In our work, the event ‘heel off’ of the gait cycle starts from the zero value of the pitch angle that follows the negative value. The event ‘toe off’ was identified by the negative peak value in one gait cycle (Figure 2).

Although any event could be chosen to define the gait cycle, in this work, it starts from one heel off the ground. If it is decided to start with heel off of the right foot, then the cycle continues until the next heel off of the same leg. The left foot goes through exactly the same series of events as the right one, but shifted in time by half a cycle.

The duration of a complete gait cycle is known as a gait cycle time, which is divided into stance time and swing time. The following terms are used to identify major events during the gait cycle:

1. Initial contact

2. Toe off

These two events divide the gait cycle into two periods, stance phase, when the foot is on the ground, and swing phase, when the foot is moving forward. The stance phase, which is also called contact phase, lasts from the initial contact to the toe off. The swing phase lasts from the toe off to the next initial contact. The angle amplitudes of initial contact (AIC) and toe off (ATO) were defined as the both limbs’ angles of initial contact and toe off, respectively.

The gait parameters were acquired from the sensors tied on feet, using the method mentioned above. Let *q*_*foot*_ represent the gesture of the foot at the very beginning of the first gait cycle after the subject was standing still on a horizontal plane, ***q***_*gait*_ represent the gesture of the foot when walking, and let both be relative to the initial coordinate system. *q*_*f-g*_ represent the quaternion of *q*_*gait*_ rotated from *q*_*foot*_. Then *q*_*f-g*_ could be acquired by:

$$q_{f-g}=q_{\mathrm{foot}}^{-1}⊗q_{\mathrm{gait}}$$

The angle between foot and the horizontal plane could be considered as the rotation angle of y-axis which corresponds to the pitch angle in Euler angles. It could be derived from *q*_*f-g*_.

### Balance level definition

Each subject performed many gait cycles in the experiment, thus the AK, AIC, and ATO of left and right limbs of all gait cycles were added respectively, to denote the angle values of each parameter (AK, AIC, and ATO). In order to quantitatively analyze the balance level of the subject, the balance levels of each parameter were defined as:

$$\mathrm{Balanc}e_{H}=\left|\frac{\mathrm{mea}n_{\mathrm{AN}}‒\mathrm{mea}n_{N}}{\mathrm{mea}n_{\mathrm{AN}}+\mathrm{mea}n_{N}}\right|$$

$$\mathrm{Balanc}e_{N}=\left|\frac{\mathrm{mea}n_{L}‒\mathrm{mea}n_{R}}{\mathrm{mea}n_{L}+\mathrm{mea}n_{R}}\right|$$

where *Balance*_*H*_, *Balance*_*N*_ represent the balance level of hemiplegic gait and normal gait, respectively, *mean*_*AN*_ and *mean*_*N*_ represent the mean values of the parameter for the abnormal and the normal limb of hemiplegic patients, respectively, *mean*_*L*_ and *mean*_*R*_ represent the mean values of left lower limb and right side of normal subjects, respectively.

## Results

### Method validation

Human body segments were considered as rigid bodies. The main strategy to analyze the motion of a rigid body was to split the motion into linear motion of the non-inertial reference point.

A database of gait cycles has been used for validation. This database included gait cycles of a group of ten normal subjects. Each subject conducted five walking trials for five meters and the gait cycles were recorded by a camera-based system. All measurement sessions were recorded using a portable video camera to count the number of gait cycles in each trial and calculate the sensitivity of the system. Then the data recorded using our method and the reference system based on the database were used to find the accuracy of the system in detection of gait and knee parameters.

**Figure 3:**
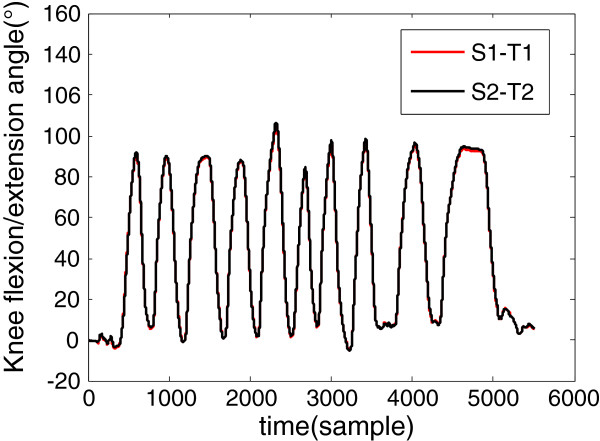
**Two knee flexion/extension angles were acquired by freedom knee flexion/extension movement**. The correlation coefficients of two curves were more than 0.9999.

A number of researches have been done on the precision and accuracy validation when using Xsens MTx for human movement assessment [24-26]. To validate the practicality of the proposed method, which does not require obtaining the accurate positions of the sensors, four sensors on the same side (two sensors on the thigh and two sensors on the shank) were used as described above, with random distances to the knee joint. The sensors on the thigh were named T1 and T2, and those on the shank were named S1 and S2. After sensor calibration, the subject was asked to perform freedom Knee flexion/extension movement. Then we obtained two knee flexion/extension angles, which were S1-T1 and S2-T2, as shown in Figure 3.

### Gait cycle and knee angle

**Figure 4:**
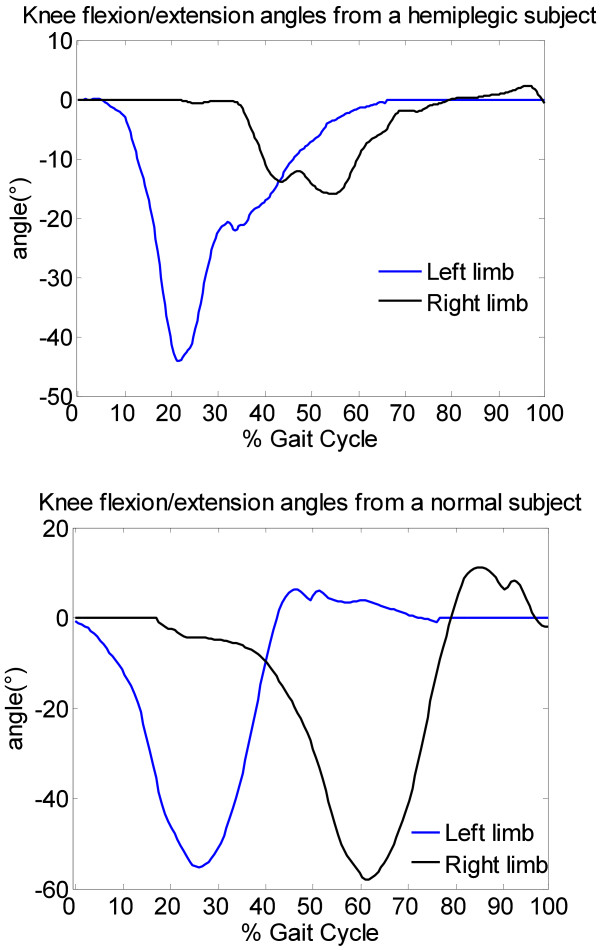
knee flexion/extension angles in degree (°) of Gait cycle from a hemiplegic subject (above) and a normal subject (below).

The angles of knee flexion/extension and the angles between foot and horizontal plane were calibrated by setting them to 0°while the subject was standing still. As shown in Figure 2, the gait cycle could be identified by the angles which were acquired from sensors on the feet. Figure 4 shows the difference of knee flexion/extension angles between hemiplegic patient and normal subject.

**Figure 5:**
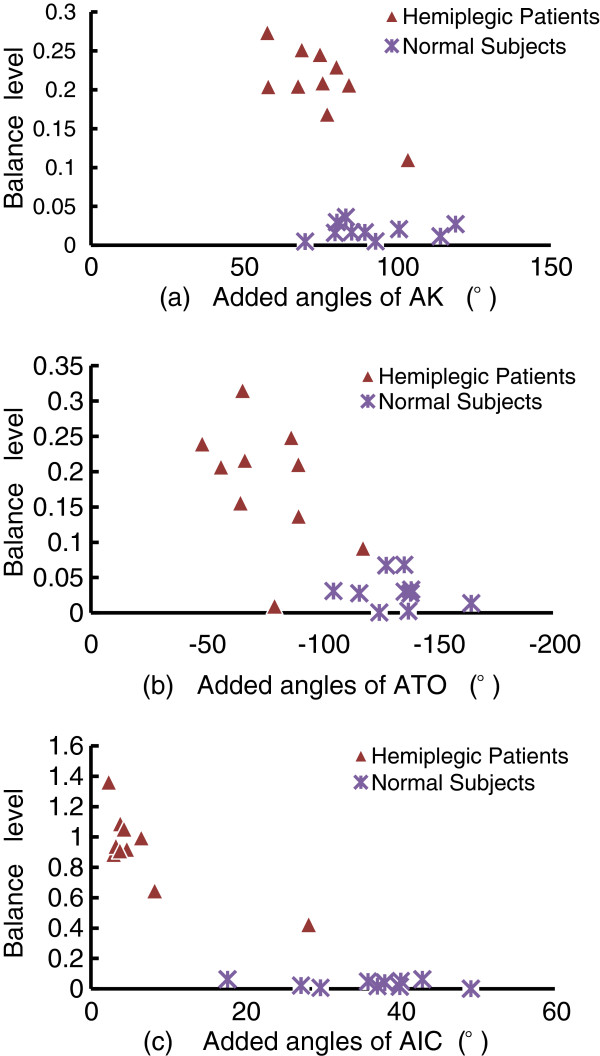
**The comparison of balance level and the added values of left and right limbs in each parameter (a. AK, b. ATO, and c. AIC) within one gait cycle.** It showed significant differences between hemiplegic gait and normal gait.

As shown in Table 1, the mean values of each parameter were calculated using ten gait cycles of each subject. Through comparison of AIC, ATO, and AK, significant differences in extensibility of lower limbs between hemiplegic patients and normal subjects were found. The absolute values of the three parameters of hemiplegic patients were significantly lower than those of normal subjects, which meant that, hemiplegic patients had worse extensibility of lower limbs compared with normal subjects. The absolute values of the three parameters of the abnormal side were also lower than those of the normal side in the hemiplegic patient group (0.94 ± 2.42 versus 4.92 ± 4.43, 29.67 ± 6.58 versus 44.91 ± 6.35, -31.6 ± 9.99 versus −44.8 ± 10.17, respectively), which means that the hemiplegic patients had worse balance of lower limbs compared with normal subjects. As shown in Figure 5(a), considering the AK parameter, the mean value of *Balance*_*H*_ was higher than the mean value of *Balance*_*N*_ (0.21 versus 0.01, p<0.01), the mean value of added angles of hemiplegic patients was lower than that of normal subjects (74.64 versus 91.31, p<0.01). In Figure 5(b), considering the ATO parameter, the mean value of *Balance*_*H*_ was higher than the mean value of *Balance*_*N*_ (0.18 versus 0.03, p<0.01), the mean value of added angles of hemiplegic patients was lower than that of normal subjects (−76.48 versus −132.4, p<0.01). In Figure 5(c), considering the AIC parameter, the mean value of *Balance*_*H*_ was higher than the mean value of *Balance*_*N*_ (0.92 versus 0.03, p<0.01), the mean value of added angles of hemiplegic patients was lower than that of normal subjects (6.77 versus 35.74, p=0.02). The higher the values of *Balance*_*H*_ and *Balance*_*n*_, the worse the balance level. It could also be concluded that the extensibility of the lower limbs of hemiplegic patients was significantly worse than that of normal subjects.

**Table 1:** Angle amplitudes (°) of Initial contact (AIC), knee flexion/extension (AK), and toe off (ATO) from hemiplegic patients (H1 TO H10) and normal subjects (N1 TO N10), “_A” represents the abnormal lower limb of the hemiplegic patients, “_N” represents the normal side of hemiplegic patients; “_L” and “_R” represent the left and right lower limb of the normal subjects, respectively

**Hemiplegic Patients**	**AIC_A**	**AIC_N**	**AK_A**	**AK_N**	**ATO_A**	**ATO_N**
H1	1.45	6.78	30.84	49.21	−39.33	−40.13
H2	0.164	2.76	32.01	45.01	−22.46	−43.17
H3	0.09	3.12	33.38	50.74	−27.29	−37.39
H4	0.183	4.44	45.9	57.38	−22.31	−33.95
H5	−0.41	2.71	20.8	36.62	−18.3	−29.8
H6	8.093	20.06	23.05	34.8	−32.55	−54.11
H7	−0.17	3.95	25.72	43.02	−35.43	−54.33
H8	0.16	3.56	28.16	46.52	−38.76	−51.1
H9	−0.11	4.38	29.88	45.68	−53.45	−64.27
H10	0.01	6.48	26.85	40.69	−26.09	−40.50
**Mean ± SD**	**0.94±2.42**	**4.92±4.43**	**29.67±6.58**	**44.91±6.35**	**−31.6±9.99**	**−44.8±10.17**
**Normal Subjects**	**AIC_L**	**AIC_R**	**AK_L**	**AK_R**	**ATO_L**	**ATO_R**
N1	13.27	13.89	49.17	51.28	−62.46	−62.36
N2	21.08	18.99	35.08	34.73	−59.59	−68.22
N3	9.39	8.27	41.78	43.23	−50.83	−54.13
N4	20.03	22.85	43.82	45.48	−69.98	−65.83
N5	19.6	20.23	40.04	43.02	−72.48	−63.7
N6	24.51	24.63	41.29	38.88	−67.22	−71.08
N7	14.96	14.73	56.30	57.65	−83.43	−81.14
N8	19.81	18.19	46.16	46.66	−66.99	−71.60
N9	17.03	18.80	61.08	57.81	−59.69	−56.44
N10	18.18	18.86	39.14	40.44	−68.51	−68.90
**Mean ± SD**	**17.79±4.10**	**17.95±4.43**	**45.39±7.65**	**45.92±7.27**	**−66.11±8.32**	**−66.28±7.43**

## Discussion

It is complicated to quantify hemiplegic gait, especially in daily life, because of the free movement. In this work, we used wearable sensors and we used only the knee angle and gait cycle, which can be easily acquired from daily life movements, to describe the differences between hemiplegic patients and normal subjects and thus to reduce the difficulty of daily monitoring of patients. The suggested method would be useful for the patient training and rehabilitation at home, providing real-time feedback about the training effect.

In daily life, various environments and long distance monitoring should be concerned. In comparison with the conventional monitoring systems based on optical camera system or magnetic position sensors, the proposed system could be used for long-term daily monitoring. Since our method is based on portable sensors and wireless communication, it allows monitoring without constraints towards location and duration.

The automated evaluation of joint and segment kinematics is valuable for clinical practice, since it can precisely measure variations and provide clarification and quantification information which cannot be obtained by manual methods of examination [27].

The method of using accelerometers, magnetometers and gyroscopes to analyze gait cycle or knee flexion/extension angle has also been used in different studies, but they were limited by the location of the sensors or drift error of the gyroscopes in long-term monitoring. The idea to use gyroscopes to assess gait was also used in different studies; however, few methods were suitable for long-term monitoring and few of them were validated by comparing to reference systems. Our new method is practical and it is not limited to the location of the sensors. It uses new signal processing approach and algorithm to acquire gait parameters.

Bohannon et al. [28,29] suggested that the ultimate goal of rehabilitation for patients with stroke is to achieve normal gait pattern and speed. Hemiplegic gait is used by clinicians to describe the pattern of limb movement and body posture of the patients with stroke [30].

**Figure 6:**
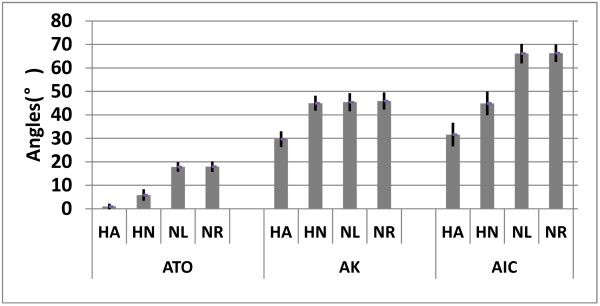
**The parameters’ absolute mean value of all hemiplegic patients and all normal subjects, respectively, which showed typical symptoms of hemiplegic gait**. “HA” represents the abnormal lower limb of hemiplegic patients, “HN” represents the normal lower limb of hemiplegic patients, “NL” and “NR” represent the left and right lower limb of normal subjects, respectively.

In this work we used initial contact, toe off and knee flexion/extension angles to acquire extensibility and balance data of lower limbs to quantitatively analyze the differences between hemiplegic gait and normal gait. Figure 6 shows the typical symptoms of hemiplegic gait:

1) Reduced angle amplitude at initial contact. This forces the foot to be in parallel with the ground before contacting it, instead contacting the ground with the forefoot. The angle amplitude is also reduced because of the decreased eccentric control of the dorsiflexors.

2) Reduced flexion/extension amplitude of knee angle caused by increased knee flexion at toe-off and during swing, due to quadriceps spasticity.

3) Decreased plantar flexion at toe-off.

Our method has several advantages over other ambulatory systems. Unlike some methods based on foot-switches or other pressure sensitive devices, no special footwear is needed for long-term monitoring, which makes this kind of monitoring more comfortable for the patient. In addition, available foot-switch-based devices limit the gait analysis to the temporal parameters [31,32], while our method provides both temporal and spatial parameters.

## Conclusions

In the present study, we examined the sagittal movements (2-D flexion-extension) of the foot and knee joint. Although the results were satisfactory and could justify the method, for real clinical application, additional experiments and analyzes are needed to find more parameters and ensure the best possible performance of the algorithm for quantitative estimation of hemiplegic gait. In addition, future extension of this study should explore the full 3-D motion of lower limbs using parameters such as step length and hip angle, since many patients with gait pathology compensate the difficulty to move the body in sagittal plane by motions in other planes.

The suggested wearable bio-motion acquisition platform provides a practical approach and it is an effective tool for discriminating gait differences between hemiplegic and asymptomatic subjects. The effect of rehabilitation training of hemiplegic patient could be quantitatively analyzed and the result could serve for real-time feedback of rehabilitation.

In this paper we just preferred to focus on the quantitative analysis of hemiplegic gait provided by our algorithm and on the applicability of the algorithm. To proper training effect feedback from the system, the real-time applicability of the algorithm is important. We intend to research and assess it in our future work.

## Abbreviations

“AIC”: represents the angle amplitude (°) of ‘Initial contact’; “ATO”: represents the angle amplitude (°) of ‘Toe off’; “AK”: represents the angle amplitude (°) of knee flexion/extension; “_A”: represents the abnormal lower limb of the hemiplegic patients; “_N”: represents the normal lower limb of the hemiplegic patients; “_L”: represents the left lower limb of the normal subjects; “_R”: represents the right lower limb of the normal subjects; “HA”: represents the abnormal lower limb of the hemiplegic patients; “HN”: represents the normal lower limb of the hemiplegic patients; “NL”: represents the left lower limb of the normal subjects; “NR”: represents the right lower limb of the normal subjects.

## Competing interests

The authors declare that they have no competing interests.

## Authors’ contributions

YG was responsible for the platform implementation, data acquisition and analysis. GZ participated in experimental design and data analysis. QL contributed to data acquisition and the article revision. ZM participated in data acquisition and data analysis. KI contributed to data analysis and article revision. LW provided the experimental infrastructure and contributed to the result discussion. All authors read and approved the final manuscript.
